# Bitter Taste Receptor T2R14 Modulates Gram-Positive Bacterial Internalization and Survival in Gingival Epithelial Cells

**DOI:** 10.3390/ijms22189920

**Published:** 2021-09-14

**Authors:** Manoj Reddy Medapati, Anjali Yadav Bhagirath, Nisha Singh, Robert J. Schroth, Rajinder P. Bhullar, Kangmin Duan, Prashen Chelikani

**Affiliations:** 1 Manitoba Chemosensory Biology Research Group, Department of Oral Biology, Rady Faculty of Health Sciences, Dr. Gerald Niznick College of Dentistry, University of Manitoba, 780 Bannatyne Avenue, Winnipeg, MB R3E 0W2, Canada; ummedapa@myumanitoba.ca (M.R.M.); Anjali.Yadav@umanitoba.ca (A.Y.B.); Nisha.Singh@umanitoba.ca (N.S.); robert.schroth@umanitoba.ca (R.J.S.); Rajinder.Bhullar@umanitoba.ca (R.P.B.); Kangmin.Duan@umanitoba.ca (K.D.); 2 Children’s Hospital Research Institute of Manitoba (CHRIM), University of Manitoba, 715 McDermot Avenue, Winnipeg, MB R3E 3P4, Canada; 3 Department of Preventive Dental Sciences, Rady Faculty of Health Sciences, Dr. Gerald Niznick College of Dentistry, University of Manitoba, 780 Bannatyne Avenue, Winnipeg, MB R3E 0W2, Canada

**Keywords:** gingival epithelial cell, G-protein-coupled receptor (GPCR), Gram-positive bacteria, host-microbe interaction, innate immunity, internalization, bitter taste receptor (T2R)

## Abstract

Bitter-taste receptors (T2Rs) have emerged as key players in host–pathogen interactions and important modulators of oral innate immunity. Previously, we reported that T2R14 is expressed in gingival epithelial cells (GECs) and interacts with competence stimulating peptides (CSPs) secreted by the cariogenic *Streptococcus mutans*. The underlying mechanisms of the innate immune responses and physiological effects of T2R14 on Gram-positive bacteria are not well characterized. In this study, we examined the role of T2R14 in internalization and growth inhibitory effects on Gram-positive bacteria, namely *Staphylococcus aureus* and *S. mutans*. We utilized CRISPR-Cas9 T2R14 knockdown (KD) GECs as the study model to address these key physiological mechanisms. Our data reveal that the internalization of *S. aureus* is significantly decreased, while the internalization of *S. mutans* remains unaffected upon knockdown of T2R14 in GECs. Surprisingly, GECs primed with *S. mutans* CSP-1 resulted in an inhibition of growth for *S. aureus*, but not for *S. mutans.* The GECs infected with *S. aureus* induced T2R14-dependent human β-defensin-2 (hBD-2) secretion; however, *S. mutans*–infected GECs did not induce hBD-2 secretion, but induced T2R14 dependent IL-8 secretion. Interestingly, our results show that T2R14 KD affects the cytoskeletal reorganization in GECs, thereby inhibiting *S. aureus* internalization. Our study highlights the distinct mechanisms and a direct role of T2R14 in influencing physiological responses to Gram-positive bacteria in the oral cavity.

## 1. Introduction

Oral epithelial cells are in constant communication with several diverse microbes under both normal and pathological conditions. However, the signaling mechanisms for pathogen recognition and innate immune responses are not yet fully understood. Host cells express toll-like receptors (TLRs), which can recognize pathogen-associated molecular patterns (PAMPs). PAMPs are bacterial cell-wall components, such as lipoteichoic acid in Gram-positive organisms and lipopolysaccharide in Gram-negative organisms [1]. Apart from these above-mentioned molecules, bacteria secrete quorum-sensing molecules (QSMs) that are recognized by bitter taste receptors (T2Rs) [2,3,4].

T2Rs belong to G-protein-coupled receptor (GPCR) superfamily of proteins and were first identified in the oral tissues [5,6]. However, several studies have shown both extra-oral expression and extra-gustatory functions for T2Rs in physiological, as well as patho-physiological, conditions [7,8]. T2Rs have now been identified as important modulators of innate immune responses in several cell types, including sinonasal epithelial cells; upper airway epithelial cells; immune cells, such as macrophages; and gingival epithelial cells (GECs) [2,9,10,11]. The stimulation of bronchial airway epithelial cells by bitter compounds resulted in an increase in intracellular Ca^2+^, resulting in increased ciliary beat frequency [12], nitric oxide (NO) production and increased bactericidal effects in sinonasal epithelial cells [10] and regulation of type-2 immunity against gut pathogens [13,14].

Apart from the epithelial cells, T2Rs are also expressed in immune cells, such as monocytes, natural killer (NK) cells, B cells, T cells and polymorphonuclear leucocytes (PMNs) [15]. Human neutrophils and phagocytes have been shown to express T2R38. T2R38 binds to bacterial QSM and AHL-12 and cause neutrophil migration [16]. In contrast, T2Rs expressed on human mast cells were not activated upon stimulation with agonists [17]. These studies suggest a tissue-specific role for T2Rs. A recent study showed that stimulation of human-monocyte-derived macrophages by agonists such as denatonium benzoate and quinine, as well as some bacterial signaling molecules, activated several T2Rs [11]. Activation of T2Rs resulted in increased NO production and enhanced phagocytosis of *Escherichia coli* and *Staphylococcus aureus* [11]. All of these studies enumerate the role of T2Rs in innate immunity. However, the physiological effect of T2R14 on Gram-positive bacteria is not well established.

We hypothesized that T2R14 activation in (GECs) results in innate immune response that is effective against Gram-positive bacteria. To test this hypothesis, we have used opportunistic pathogen *S. aureus*, which is often associated with oral disorders, such as mucositis, angular cheilitis and parotitis. We also followed up on our recent studies, where we analyzed the effects of the competence stimulating peptides (CSPs) secreted by the cariogenic *S. mutans* in GECs [2,18]. *S. mutans* CSP-1 is shown to activate T2R14, leading to an innate immune response in GECs. However, the underlying mechanisms of these innate immune responses and physiological effects of T2R14 on Gram-positive bacteria are not characterized. Therefore, one of the major questions we answered in this study is whether this innate immune response is deleterious for *S. mutans* or is *S. mutans*, using the host immune system to inhibit other Gram-positive bacteria. The CSP-1- and bitter-agonist-mediated T2R14 activation in GECs inhibits growth of *S. aureus* but not for *S. mutans*. Further, our results suggest that T2R14 influences the cytoskeleton rearrangement and effects internalization of Gram-positive bacteria in GECs.

## 2. Results

### 2.1. T2R14 Mediates Internalization of S. aureus and S. mutans in GECs

A recent study on macrophages suggests that T2Rs are one of the important receptors in the phagocytic machinery [11]. Although immune cells are the primary mediators of bacterial phagocytosis, epithelial cells in the oral cavity and the upper respiratory tract are the first line of defense against microbes. These epithelial cells are also known to internalize several species of bacteria and also express several T2Rs. However, the role of T2Rs in internalization of bacteria, especially Gram-positive pathogens, such as the cariogenic *S. mutans* and the opportunistic pathogen *S. aureus*, is not studied in GECs. To characterize the role of T2R14 in bacterial internalization, the OKF6 WT, MOCK and T2R14 KD cells were infected by using *S. aureus* and *S. mutans*.

**Figure 1:**
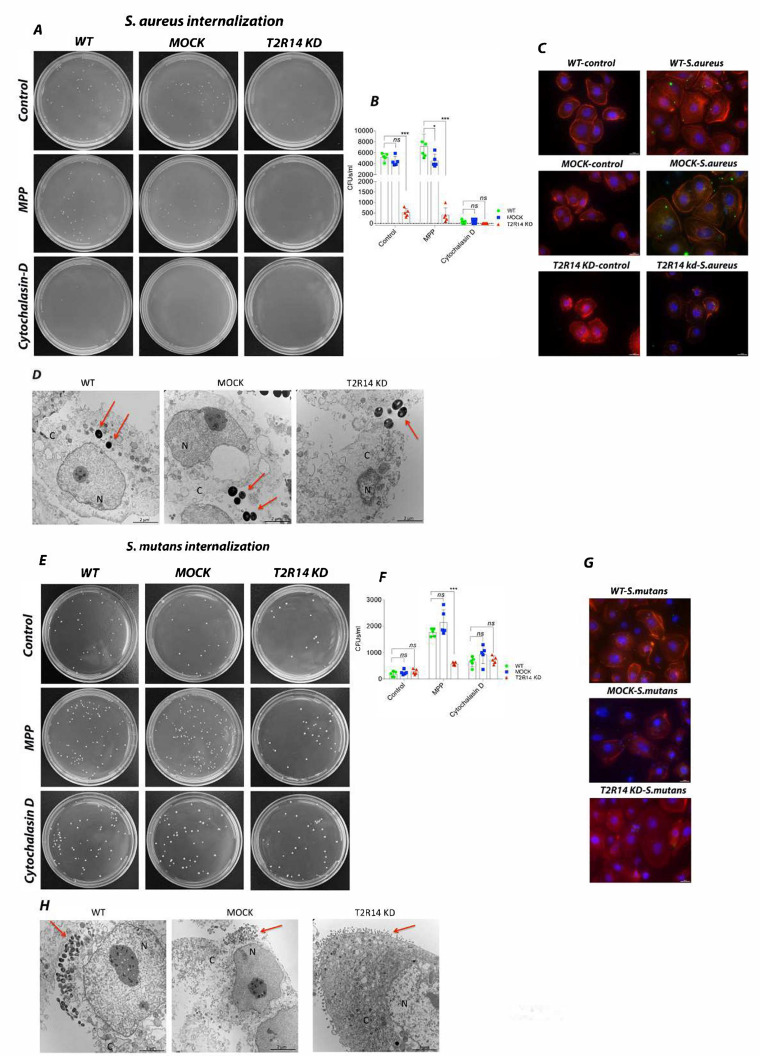
Internalization of *S. aureus* and *S mutans* in OKF6 WT, MOCK and T2R14 KD cells. (**A**) Representative LB agar plates depicting *S. aureus* colonies that are internalized in OKF6 WT, MOCK and T2R14 KD cells pretreated with MPP (5 μM) and cytochalasin D (1 μg/mL). A total of 100 μL of the diluent containing bacteria was grown on LB agar plates for 24 h to form CFUs. (**B**) *S. aureus* CFUs from representative LB agar plates were calculated and represented in a grouped bar graph. (**C**) Immunofluorescence (IF). OKF6 cells infected with BCECF-AM (10 μM) labelled *S. aureus*. Cells were fixed with paraformaldehyde and labeled with TRITC-phalloidin (50 μM) and DAP1 (1:10,000 dilution). The IF image shows nucleus stained with DAPI (blue), F-actin stained with TRITC-phalloidin (red) and *S. aureus* stained with BCECF-AM (green). The representative images are from 3 independent experiments. (**D**) Transmission electron microscopy (TEM) images of *S. aureus* internalized OKF6 WT, MOCK and T2R14 KD cells. Nucleus (N), cytoplasm (C) and the red arrows in the image point to *S. aureus.* Scale = 2 microns. The representative images are from 1 independent experiment and captured from 5 different fields. (**E**) Representative BHI agar plates depicting *S. mutans* colonies that are internalized in OKF6 cells pretreated with MPP (5 μM) and cytochalasin D (1 μg/mL). A total of 100 μL of the diluent containing bacteria was grown on BHI agar plates for 24 h to form CFUs. (**F**) *S. mutans* CFUs from the representative BHI agar plates were calculated and represented in a grouped bar graph. The data represented in the graphs are SEM of ≥5 independent experiments. Two-way ANOVA analysis, using Tukey’s multiple comparison analysis, was performed, and the observed *p*-value is *** *p* = 0.002. (**G**) The IF image shows OKF6 cells infected with BCECF-AM (green) labeled *S. mutans*, and nucleus stained with DAPI (blue) and TRITC-phalloidin (red). The IF images were captured using a Nikon Ti microscope using 60X oil immersion objective. The representative images are from 3 independent experiments. (**H**) TEM images of *S. mutans* internalized OKF6 WT, MOCK and T2R14 KD cells. The red arrows in the image point to *S. mutans.* The representative images are from 1 independent experiment and captured from 5 different fields.

In OKF6 T2R14KD cells, the internalization of *S. aureus* was significantly decreased as compared to the WT and MOCK cells, as represented by the colony-forming units (CFUs) (Figure 1A,B) and confirmed by immunofluorescence (Figure 1C) and TEM (Figure 1D). When compared to control group, the GECs pretreated with TLR signaling inhibitor (MPP) did not show any changes in the pattern of internalization between WT, MOCK and T2R14 KD GECs. However, the internalization levels decreased significantly in WT and MOCK cells treated with cytochalasin D (Figure 1A,B).

Next, we characterized the internalization of cariogenic bacteria *S. mutans* in WT, MOCK and T2R14 KD GECs. In the control group, there was no significant change in internalization between WT, MOCK and T2R14 KD GECs. In the MPP-treated group, the internalization of *S. mutans* significantly increased in WT and MOCK cells, but not in T2R14 KD GECs. In the cytochalasin-D-treated group, the internalization of *S. mutans* is similar between WT, MOCK and T2R14 KD GECs (Figure 1E–H).

### 2.2. Knockdown of T2R14 Decreases PAK1 Associated Actin and F-Actin but Not GTP-Rac1 in GECs

**Figure 2:**
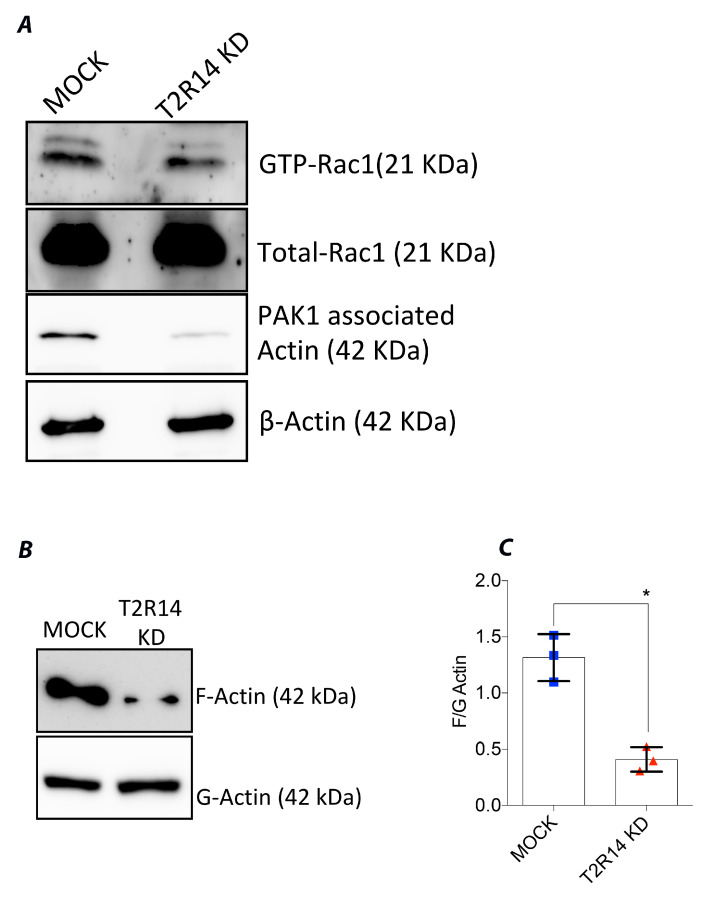
Characterizing the GTP-Rac1, F/G actin levels in MOCK and T2R14 KD OKF6 cells. (**A**) The MOCK and T2R14 KD OKF6 cell lysates (500 μg) were incubated with PAK1 agarose beads (20 μg) for 1 h at 4 °C. After incubation the beads were washed, re-suspended in 2× Laemmli buffer and separated by 12% SDS-PAGE. Western blot analysis of PAK1 pull-down and total protein lysates using primary mouse monoclonal anti-Rac1 antibody (1:1000), mouse monoclonal anti β-Actin antibody (1:25,000) and secondary anti-mouse HRP antibody (1:5000 and 1:25,000) re-spectively. To visualize GTP-Rac1 that is at low levels compared to Total-Rac1, the blots were exposed for 5 min. To visualize PAK1 associated Actin and β-Actin the blots were exposed for 1 min. (**B**) Western blot analysis of F and G actin fractions isolated from MOCK and T2R14 KD cells and separated by 10% SDS-PAGE. The blots were probed using primary mouse monoclonal anti β-Actin antibody and exposed for 1 min. (**C**) The corresponding densitometry analysis of F and G actin isoforms. The data represented in the graphs are SEM of ≥3 independent experiments. One-way ANOVA analysis, using Tukey’s multiple comparison analysis, was performed, and the observed p-value is * *p* < 0.05. The blots were developed by using chemiluminescence detection system and imaged by using a ChemiDoc MP imaging system, Bio-Rad, Toronto, ON, Canada).

The canonical internalization mechanism involves activation of the Src pathway, leading to cortactin recruitment, followed by the polymerization of actin [19,20,21]. The actin polymerization and cytoskeletal rearrangements are controlled by small GTPases, especially Rac1. To characterize the role of these GTPases and their relationship to T2R14 activation, pull-down assays were performed in MOCK and T2R14 KD GECs. The MOCK and T2R14 KD GECs exhibited similar levels of GTP-Rac1 (first panel, Figure 2A). However, in T2R14 KD GECs, there is a decreased pull-down of PAK1 associated actin (third panel, Figure 2A). To characterize the T2R14-mediated actin polymerization, G and F actin isoforms were isolated in MOCK and T2R14 KD GECs. The Western blot analysis on MOCK and T2R14 KD GECs shows a decreased level of F-actin upon T2R14 KD (Figure 2B,C).

### 2.3. S. aureus–Induced hBD-2 Secretion Is T2R14 Dependent

**Figure 3:**
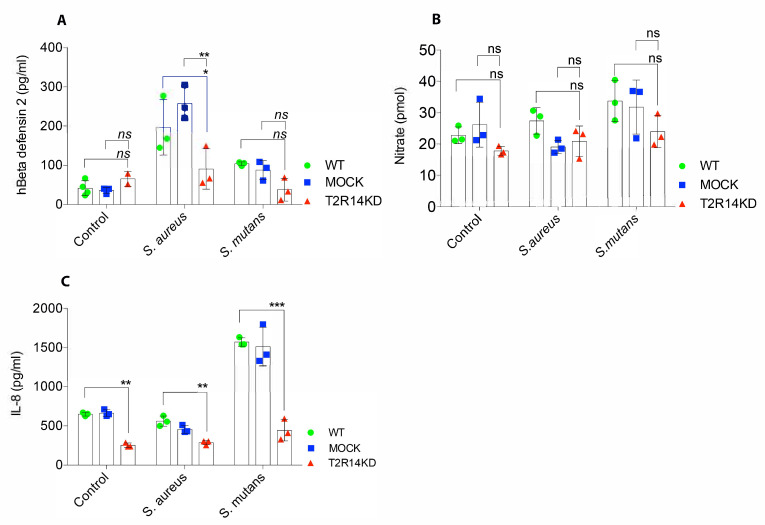
Characterizing the secretion of antimicrobial peptide hBD-2, nitrite/nitrate and IL-8/CXCL8 by ELISA. The OKF6 WT, MOCK and T2R14 KD cells were infected with *S. aureus* (50 MOI) and *S. mutans* (100 MOI) for 18 h. After infection, the supernatants were collected and filtered by using 0.25 μm nylon filter to remove cellular debris and bacteria. The supernatant was then used to determine secreted innate immune markers, such as (**A**) hBD-2, (**B**) nitrite/nitrate and (**C**) IL-8/CXCL8 as mentioned in methods. The representative grouped bar graphs were generated by using GraphPad Prism 7.0. The data represented in the graphs are SEM of ≥3 independent experiments. Two-way ANOVA analysis, using Tukey’s multiple comparison analysis, was performed, and the observed *p*-values are * *p* = 0.01, ** *p* = 0.004 and *** *p* = 0.002, ns = not significant.

Defensins are small cationic peptides that are known to exert antimicrobial responses towards both Gram-negative and Gram-positive bacteria by destroying their cell envelope [22]. To characterize the role of T2R14 in hBD-2 secretion, the OKF6 WT, MOCK and T2R14 KD cells were infected with *S. aureus* and *S. mutans* for 18 h. Post-infection, the conditioned medium (CM) from WT and MOCK cells infected with *S. aureus* showed a significant increase in hBD-2 levels as compared to the T2R14 KD GECs. Interestingly, WT, MOCK and T2R14 KD GECs infected with *S. mutans* did not show any significant change in the secretion of hBD-2. The basal levels of hBD-2 between WT, MOCK and T2R14 KD GECs remain unaffected (Figure 3A). These data suggest that *S. aureus* and *S. mutans* elicit differential hBD-2 secretion in the GECs.

### 2.4. Gram-Positive Bacteria Do Not Induce Nitrite/Nitrate Secretion in GECs

Epithelial cells produce nitric oxide (NO) as a part of innate immune response against bacteria. Previous studies have shown a T2R-mediated increase in NO upon treatment with Gram-positive bacteria, such as *Staphylococcus epidermidis* and *Bacillus cereus* [9,23]. However, the T2R-mediated NO secretion in oral epithelial cells has not been characterized thus far. To characterize the T2R14-mediated NO secretion, OKF6 WT, MOCK and T2R14 KD cells were infected with *S. aureus* and *S. mutans*, respectively. After infection, the supernatants were collected and the end products of NO (nitrite/nitrate) were measured by fluorometric method, using a reference standard. The results from these assays show that OKF6 cells secrete basal levels of nitrite/nitrate and these levels remain unchanged upon infection with Gram-positive bacteria, namely *S. aureus* and *S. mutans* (Figure 3B).

### 2.5. IL-8/CXCL-8 Secretion in GECs Is T2R14-Dependent

Oral epithelial cells are known to secrete IL-8 chemokine, a potent neutrophil attractant upon bacterial insult. Previously, we have shown an increased IL-8 secretion in GECs upon treatment with T2R14 agonists, including *S. mutans* CSP-1, as well as a decreased secretion of IL-8 upon knockdown of T2R14 [2]. However, T2R14 dependent IL-8 secretion profile upon bacterial insult has not been investigated.

To analyze the secretion of IL-8 upon bacterial insult, OKF6 WT, MOCK and T2R14 KD cells were infected with *S. aureus* and *S. mutans* for 18 h. The analysis of CM from these cells showed a significant decrease in basal IL-8 secretion in T2R14 KD GECs compared to WT and MOCK cells. Interestingly, the levels of IL-8 did not change upon infection with *S. aureus*, while the infection of WT and MOCK cells with *S. mutans* significantly decreased IL-8 secretion (Figure 3C).

### 2.6. Activation of T2R14 in GECs by CSP-1 and DPH has a Growth Inhibitory Effect on S. aureus but Not S. mutans

**Figure 4:**
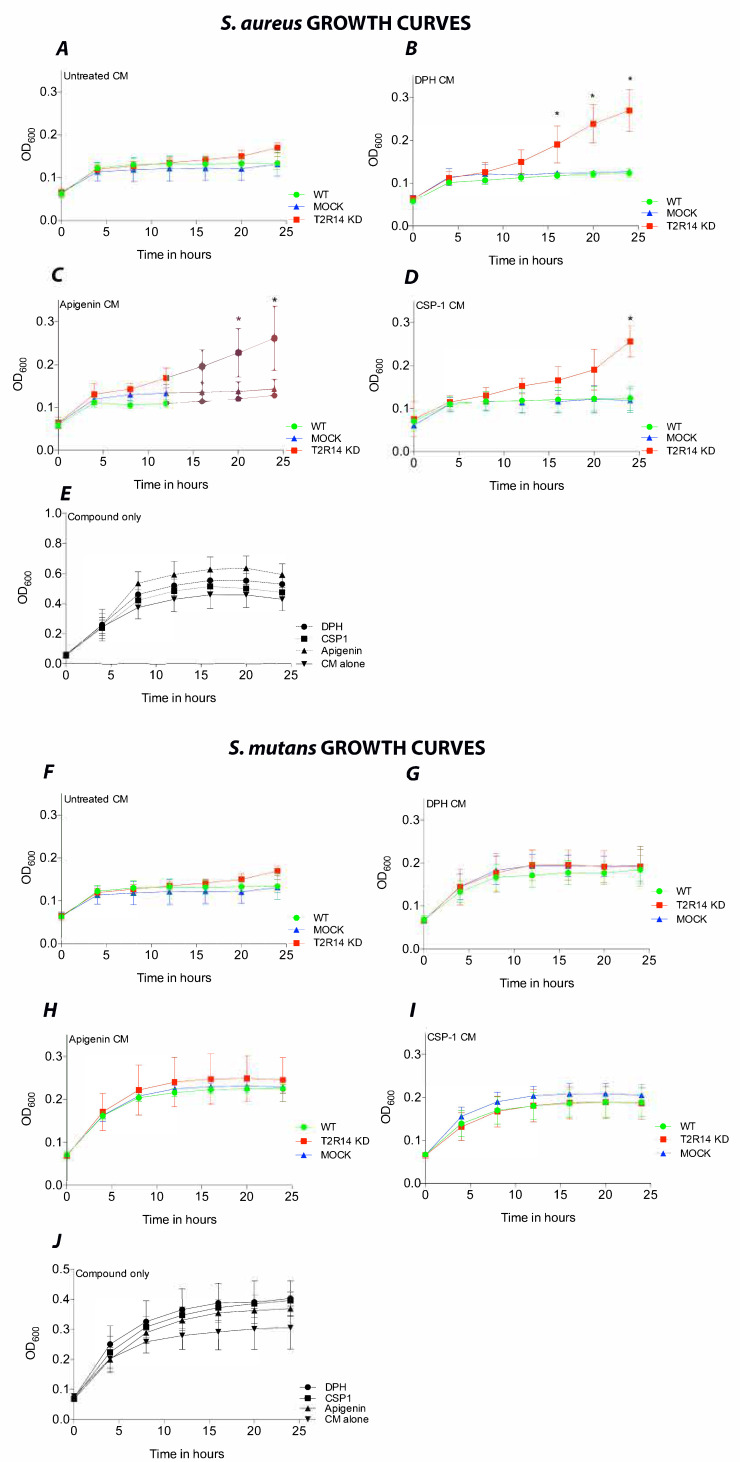
Characterizing the bactericidal effect of T2R14 on *S. aureus* and *S. mutans*. Real-time analysis of bacterial growth over 24 h measured at OD_600_. (**A**–**E**) Real-time growth curves of *S. aureus* in response to WT, MOCK and T2R14 KD CM alone or with T2R14 agonists DPH (250 μM), CSP-1 (50 μM), apigenin (50 μM) and compound alone treatment. (**F**–**J**) Real-time growth curves of *S. mutans* in response to WT, MOCK and T2R14 KD CM alone or with T2R14 agonists DPH (250 μM), CSP-1 (50 μM), apigenin (50 μM) and compound alone treatment. The data represented in the graphs are SEM of ≥3 independent experiments. One-way ANOVA analysis, using Tukey’s multiple comparison analysis, was performed, and the observed *p*-value is * *p* < 0.05.

To characterize the bacterial growth inhibitory effect of T2R14, the OKF6 WT, MOCK and T2R14 KD cells were treated with the recently identified T2R14 agonist CSP-1 secreted by *S. mutans* [2], and bitter agonists DPH and apigenin. The CM from the abovementioned treated GECs was used to test the bactericidal effect on *S. aureus* and *S. mutans.* The results show that CM from untreated WT, MOCK and T2R14 inhibited the growth of *S. aureus* (Figure 4A). The CM from DPH-treated T2R14 KD GECs appears to have rescued *S. aureus* growth, as observed in the real-time growth curve (Figure 4B). The CM from CSP-1- and apigenin-treated T2R14 KD GECs rescued *S. aureus* growth, as is evident in the real-time growth curves when compared to WT- and MOCK-treated cells (Figure 4C,D). The T2R14 agonists alone did not inhibit *S. aureus* growth (Figure 4E).

Next, we characterized the growth inhibitory effect of T2R14 activation on cariogenic bacterium *S. mutans*. The CM from untreated WT, MOCK and T2R14 KD GECs inhibited the growth of *S. mutans* (Figure 4F). However, none of the T2R14 agonists, including CSP-1, had any noticeable effect on *S. mutans* growth (Figure 4G–I). The growth of bacteria in untreated and those treated with agonists remained the same. The T2R14 agonists alone did not inhibit *S. mutans* growth (Figure 4J).

## 3. Discussion

T2Rs have recently emerged as important receptors in innate immunity. Studies suggest different bacterial metabolites and QSMs to be interacting and signaling through several T2Rs [2,3,4]. The T2R–QSM interactions are shown to induce several innate immune responses, such as NO secretion mainly in the sinonasal cavity, upper airway epithelial cells and in macrophages [9,11,24]. Although these findings provide insightful knowledge into T2R-mediated innate immune mechanisms, there exists a gap in knowledge mainly in deciphering the physiological effects of T2Rs on bacteria. In this context, our study addresses the physiological effects of T2R14 on Gram-positive pathogenic bacteria, namely *S. aureus* and *S. mutans*.

Our results show that *S. aureus*, but not *S. mutans*, internalization is significantly decreased upon T2R14 KD in GECs (Figure 1B,F). Our results show that, upon inhibition of actin polymerization, the internalization of *S. aureus* is significantly decreased in WT, MOCK and T2R14 KD GECs. Interestingly, the TLR signaling inhibitor MPP did not have effect on the internalization of *S. aureus* but inhibited *S. mutans* internalization in combination with T2R14 KD. Previous studies have shown that *S. aureus* is known to internalize via F-actin-mediated host-directed endocytosis [20]. The *S. aureus* internalization was completely abolished in WT, MOCK and T2R14 KD upon treatment with cytochalasin D (Figure 1A,B), and T2R14 KD GECs exhibited decreased levels of F-actin (Figure 2B,C). The data suggest that T2R14 promotes F-actin, which in turn mediates internalization of *S. aureus* in GECs. It is possible that knockout of T2R14 can lead to changes in the protein expression of *S. aureus*–binding proteins on the host cell membranes, such as α5β1 integrins, cortactins and FAK, leading to a decrease in *S. aureus* internalization. Further studies into the analysis of expression profiles of the abovementioned proteins in T2R14 KD cells may shed light on this mechanism.

To better understand the molecular mechanism behind T2R14-regulated bacterial internalization, we performed small GTPase activation assays for Rac1, and quantified F-actin levels upon T2R14 KD. Actin cytoskeleton reorganization facilitates bacterial internalization and endocytosis. Our results indicate decreased PAK1-associated actin pull-down and decreased F-actin levels in T2R14 KD GECs compared to MOCK cells. Previously, we showed that bitter-agonist-quinine-mediated activation of T2R4 inhibits Rac1 activation [25]. Hence, there exists a link between T2Rs and small GTPases and their possible role in cytoskeletal reorganization. Previous studies on platelet cells have shown PAK1 (GTP-Rac1 binding partner) to interact with cortical-actin-binding protein (cortactin) and N-WASP, which interact with actin [26,27]. However, in GECs, the effect of T2R14 KD on expression of cortactin and N-WASP and their interaction with PAK1 remains to be determined.

T2Rs have emerged as important receptors in modulating innate immune responses, and their activation is known to induce protective responses mainly in upper airway epithelial cells. We characterized T2R14-mediated innate immune responses, such as hBD-2, NO and IL-8, upon infection of GECs with *S. aureus* and *S. mutans*. Interestingly, only WT and MOCK cells treated with *S. aureus* secreted higher levels of hBD-2, but not T2R14 KD GECs. Recent study of gingival tissues of gustducin knockout mice showed decreased levels of beta-defensins and LL-37 [28]. Intriguingly, in our study, *S. mutans* treatment did not elicit significant hBD-2 response in the presence or absence of T2R14 (Figure 3A). A recent study utilizing primary GECs also reported minimal secretion of hBD-2 upon stimulation with *S. mutans* [29]. It is not surprising to observe that only certain bacterial species induce AMP secretions. While *S. aureus* is a low-abundance commensal, infection with *S. aureus* is often associated with mucositis, angular cheilitis and parotitis. An earlier study showed differential hBD-2 regulation in oral keratinocytes in response to pathogenic and commensal bacterial species [30]. Hence, in-depth analysis of other AMPs in the presence of different bacterial strains is warranted.

**Figure 5:**
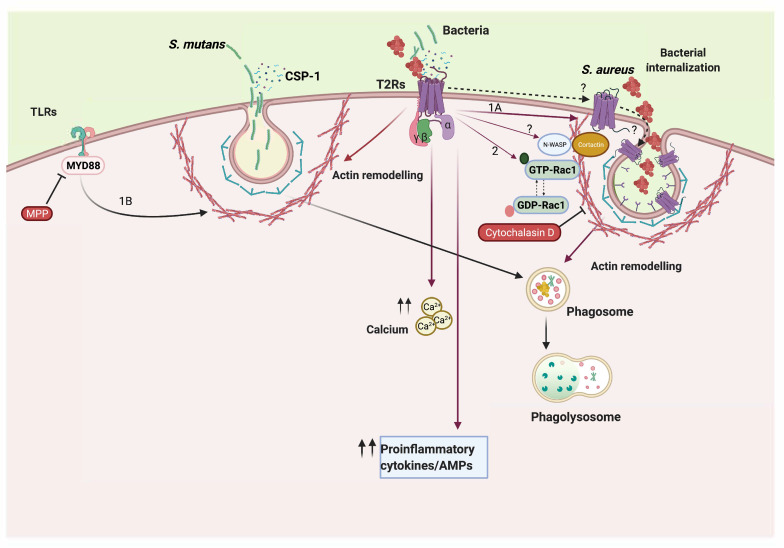
Schematic showing the mechanism of T2R-mediated Gram-positive bacterial internalization and survival in GECs. T2R14-mediated internalization of *S. aureus* might involve actin remodeling, while *S. mutans* internalize via T2R/TLR-dependent mechanisms in GECs (pathway (1A,B)). T2R14 in GEC influences actin remodeling (based on cytochalasin D treatment and PAK1-associated actin pull-down), thus modulating bacterial internalization (pathway (2)). The activation to T2R14 signaling by bacteria or their signaling molecules leads to an increased secretion of innate immune markers, such as IL-8/CXCL8 and antimicrobial peptide hBD-2 in GECs. It is currently unknown whether T2R14 internalizes into clathrin-coated vesicles and signals (dotted arrows on the membrane). Figure was drawn by using BioRender, Toronto, ON, Canada.

The gingival epithelial barrier is the first line of defense against several pathogenic bacteria. In response to increasing numbers of bacteria, it is known to secrete several cytokines and chemokines, such as IL-8/CXCR8, to attract neutrophils and monocytes to the site of infection. In our previous study, we showed that T2R14 agonist and CSP-1 from *S. mutans* induce the secretion of IL-8, and this is abolished upon a knockdown of T2R14 leading to decreased neutrophil chemotaxis [2]. In the present study, we observed a similar effect in which T2R14 expressing GECs were able to secrete significantly higher amounts of IL-8 upon infection with *S. mutans* as compared to the T2R14 KD cells (Figure 3C). Although the LPS from Gram-negative bacteria and *S. mutans* are known to signal via TLRs, the regulation of IL-8 by T2R14 KD can affect the TLR signaling responses. Hence, our findings point to a possible crosstalk between T2R and TLR signaling pathways (Figure 5). This interesting finding highlights the importance of T2R14 in regulating innate immune responses in oral epithelial cells.

Finally, we have performed bacterial growth inhibition assay to characterize the physiological role of T2R14 in GECs. Previously, in SCCs the bitter agonist denatonium induced AMP secretion and invoked bactericidal effect on Gram-negative CF bacterium *Pseudomonas aeruginosa* [31]. Our data show that DPH, CSP-1 and apigenin CM from T2R14 KD cells increased *S. aureus* growth compared to that of WT and MOCK. Intriguingly, the CM from untreated cells inhibited *S. aureus* growth and addition of bitter agonist blunted the inhibitory response only in T2R14 KD cells. However, we did not observe similar effects on *S. mutans*. Our data reflect previous studies in which inhibition of Gram-negative bacteria, such as *P. aeruginosa*, were mediated by T2Rs agonists, such as denatonium [31,32]. In our cell model, the bactericidal effect was minimal compared to effect induced by denatonium in sinonasal ciliary cells [31,32]. However, the effect we observed in GECs is comparable to that in sinonasal ciliary cells in which lower concentrations of bitter agonist (0.1 mM denatonium) had a minimal bactericidal effect compared to higher concentrations of agonist (10 mM denatonium). In our study, lower concentrations of bitter agonists and QSMs were chosen to better reflect the physiological concentrations. While the differential bactericidal effects on *S. aureus* and *S. mutans* are interesting, one possible explanation could be the high biofilm formation by the strain used in this study, *S. mutans* UA159, thus evading host response mechanisms [33]. Another interesting observation is the inhibition of *S. aureus* upon treatment of GECs with CSPs from *S. mutans*. This result is in agreement with a recent study that isolated bioactive molecules from biofilms of *S. mutans* and observed a bactericidal effect on other commensal bacteria [34]. Our finding provides hints into a host-directed mechanism by which bacteria, such as *S. mutans*, overcome competition by other commensals, such as *S. aureus*, in dysbiotic states (Figure 5).

## 4. Materials and Methods

### 4.1. Reagents Used in the Study

Methylpiperidino pyrazole (MPP) (#13863), Apigenin (#10010275) and nitrate/nitrite fluorometric assay kit (#780051) were purchased from Cayman Chemical Company, Ann Arbor, MI, USA. Cytochalasin D (#C8273), TRITC-Phalloidin (#P1951) and Diphenhydramine hydrochloride (#PHR1015) were purchased from Sigma Aldrich, Oakville, ON, Canada. The 2’,7’-Bis-(2-Carboxyethyl)-5-(and-6)-Carboxyfluorescein-Acetoxymethyl Ester (BCECF-AM) (#B1170) was purchased from Thermo Fisher Scientific, Carlsbad, CA, USA. Synthetic *S. mutans* competence stimulating peptide (CSP-1) of 98% purity was purchased from GenScript (Picataway, NJ, USA). Human β-defensin 2 (hBD-2) ELISA kit (#900-K172) was purchased from PeproTech, Cranbury, NJ, USA and IL-8/CXCL8 ELISA kit (#D8000C) was purchased from R&D systems, Toronto, ON, Canada. Keratinocyte growth medium-2 (KGM-2) for OKF6 cell culture was purchased from Promo Cell (Heidelberg, Germany). The following antibodies were purchased: mouse monoclonal anti-β-actin (#A5441) from Sigma Aldrich (Oakville, ON, Canada), goat anti-rabbit IgG-HRP conjugate (#17-6515) from Bio-Rad (Mississauga, ON, Canada), goat anti-mouse IgG-HRP conjugate (#A-10668) and rabbit polyclonal anti-T2R14 (#OSR00161W) from Thermo Fischer Scientific, Carlsbad, CA, USA, and mouse monoclonal anti-Rac1 (#05-389) from Millipore, Oakville, ON, USA.

### 4.2. Cell Line Used in the Study

The oral keratinocyte cell line OKF6 was a kind gift from Dr. Gill Diamond, University of Florida [35,36]. The T2R14 KD and a non-targeting (MOCK) Alt^®^-R-control CRISPR-crRNA or MOCK OKF6 cells were generated by using the CRISPR-Cas9 technique [37], and published in our previous study [2]. The expression of T2R14 in WT, MOCK and T2R14 KD cells is confirmed by Western blot analysis and flow cytometry analysis (Supplementary Materials Figure S1). The protocols for Western blot detection and flow cytometry analysis of T2R14 was previously described [2].

### 4.3. Bacterial Strains Used in the Study

The *S. aureus* (ATCC strain 6538) and *S. mutans* strain UA159 were purchased from ATCC. The *S. aureus* strain is propagated in Luria-Bertani (LB) broth at 37 °C, under constant agitation. The *S. mutans* strain is propagated in Brain Heart Infusion (BHI) broth at 37 °C and 5% CO_2_, under constant agitation.

### 4.4. Bacterial Internalization Assay

For internalization assay, *S. aureus* and *S. mutans* were grown overnight at 37 °C, under constant agitation, in the corresponding liquid medium. The early log-phase cultures for both *S. aureus* and *S. mutans* were used for infections. For infection with *S. aureus*, an MOI of 50:1 (50 bacteria per 1 epithelial cell), and for *S. mutans*, an MOI of 100:1 (100 bacteria per 1 epithelial cell) were resuspended in GEC medium [38,39]. The OKF6 WT, MOCK and T2R14 KD cells were infected with *S. aureus* for 1 h and with *S. mutans* for 2 h at 37 °C [38,39]. These conditions were optimized prior to the experiments, and the best MOI and infection times were chosen based on cell-viability assays. After infection, the cells were washed four times with sterile PBS. The extracellular bacteria were killed by treating the cells with gentamycin (100 μg/mL) for 1 h. After antibiotic treatment, the cells were washed four times, using sterile PBS, and were incubated with 1% saponin for 15 min [40]. Then, 100 μL of lysate was spread on LB agar and BHI plates and incubated for 24 h at 37 °C. After 24 h, the plates were removed, and then colonies were counted manually and represented in colony forming units (CFUs).

### 4.5. Immunofluorescence Microscopy of Internalized Bacteria in Host Cells

The log-phase cultures of *S. aureus* and *S. mutans* were labeled for 45 min with 2’,7’-Bis-(2-Carboxyethyl)-5-(and-6)-Carboxyfluorescein-Acetoxymethyl Ester (BCECF-AM) (10 μM) [40]. BCECF-AM is a non-fluorescent membrane permeable dye that is converted to fluorescein (BCECF) by intracellular esterases. The labeled bacteria were washed, using sterile PBS, and re-suspended in GEC medium. The OKF6 WT, MOCK and T2R14 KD cells were infected by using BCECF-AM-labeled *S. aureus* (50 MOI for 1 h) and *S. mutans* (100 MOI for 2 h). After infection, the GECs were washed three times, using PBS, and were fixed, using 4% paraformaldehyde. After fixation, GECs were washed three times, using PBS, and the actin filaments were labeled, using TRITC-phalloidin (50 μM) for 45 min, followed by washing thrice with PBS. The nucleus was stained with DAPI (1:10,000 dilution) for 5 min. The cover slips were then mounted onto a glass slide, using Fluoromount G mounting medium (Southern Biotech, Birminghan, AL, USA). The cells were imaged by using a Nikon Eclipse Ti microscope, using narrow standard band-pass filters for DAPI, FITC for BCECF-AM and Texas Red for TRITC-phalloidin. The images were acquired at different focal points, using a Z-stack program. In total, 30 stacks were obtained, with each stack having a size of 0.3 μm. The 30-stack image was then processed to correct for out-of-focus light by deconvolution, using a wide-field point spread function algorithm in NIS-elements software.

### 4.6. Transmission Electron Microscopy Imaging of GECs

The OKF6 WT, MOCK and T2R14 KD cells treated with *S. aureus* and *S. mutans* were fixed with 3% gluteraldehyde in 0.1 M Sorensen’s buffer for 3 h. After fixation the cells were suspended in sucrose solution and stored at 4 °C for processing. The above fixed cell suspensions were embedded into plastic resins and thin sections (90–100 nm) are placed on mesh copper grids. The copper grids are finally stained with osmium tetroxide and uranyl acetate. The grids were imaged by using a Philips CM10 microscope at a magnification of 10,500×.

### 4.7. GTP-Rac1 Pull-Down Assay

The OKF6 WT, MOCK and T2R14 KD cells were lysed, and the total protein was extracted according to the manufactures protocol (#BK030, cytoskeleton, Denver, CO, USA). The protein concentration was quantified, and 500 μg of total protein was incubated with 50 μg of PAK1 beads for 1 h at 4 °C. After 1 h of incubation, the beads were centrifuged at 5000× *g* at 4 °C for 1 min. The supernatant was carefully removed, and the beads were washed, using wash buffer, and centrifuged at 5000× *g* at 4 °C for 3 min. The wash buffer was carefully discarded, and 2× Laemmli buffer was added to the beads. The sample was then boiled for 2 min, Western blot analysis was performed and the membranes were probed with anti-Rac1 and anti-actin antibodies.

### 4.8. Isolation and Quantification of F/G-Actins in GECs

Briefly, the OKF6 cells were homogenized in cold lysis buffer (10 mM K_2_HPO_4_, 50 mM KCL, 100 mM NAF, 2 mM MgCl_2_, 1 mM EGTA, 0.2 mM DTT, 0.5 Triton X-100 and 1mM sucrose, at pH 7.9. The soluble actin (G-actin) was centrifuged at 15000× *g* for 30 min. The insoluble actin (F-actin) was suspended by lysis buffer and equal volume of buffer 2 (1.5 mM guanidine hydrochloride, 1 mM sodium acetate, 1 mM CaCl_2_, 1 mM ATP and 20 mM Tris-HCl, at pH 7.5) incubated on ice for 1 h after which the samples were centrifuged at 15,000× *g* for 30 min, and F-actin was measured in this supernatant. The fractions were analyzed by Western blotting.

### 4.9. Bacterial Survival Assay

The early log-phase cultures of *S. aureus* and *S. mutans* were used for the survival assay. The bacteria were centrifuged at 5000× *g* for 5 min, and the pellet was resuspended with conditioned medium (CM) from OKF6 WT, MOCK and T2R14 KD cells treated with DPH, CSP-1 and apigenin. For real-time analysis, the bacterial pellet was resuspended in 100 μL of CM and was plated in a clear-bottom 96-well plate layered with sterile white mineral oil to prevent evaporation of the media. The OD_600_ was measured every 10 min for a period of 24 h, using a Synergy H4 Multimode Microplate Reader (BioTek, Winooski, VT, USA).

### 4.10. Human β-Defensin 2 (hBD-2) ELISA

Briefly, OKF6 WT, MOCK and T2R14 KD cells were seeded in a 12-well plate at a density of 1.5 × 10^5^ cells/well. These cells were infected with *S. aureus* and *S. mutans* for 18 h at an MOI of 50 and 100, respectively. After the treatments, supernatant was collected and filtered by using 0.2 μm nylon filter. The filtered supernatant was used to determine the secreted levels of hBD-2, using ELISA (# 900-K172, PeproTech, Cranbury, NJ, USA). The detection limit of the assay is (1500–23 pg/mL). The absorbance A_450_ nm was measured by using a Flexstation3 plate reader (Molecular Devices, San Jose, CA, USA).

### 4.11. Measurement of Nitrate/Nitrite Secretion

The supernatant from OKF6 WT, MOCK and T2R14 KD cells was collected, and 50 μL was used to measure the secreted levels of nitrate/nitrite, using a fluorescent assay kit (#780051, Cayman Chemical, Ann Arbor, MI, USA). The reductase enzyme cofactor and reductase enzyme were mixed with supernatant and incubated for 30 min at RT followed by addition of 2,3-diaminoapthelene (DAN) and NaOH. The detection limit of the assay is 500–7.8 pmol. The fluorescence was measured at an excitation wavelength of 365 nm and an emission wavelength of 430 nm, using a Flexstation3 plate reader (Molecular Devices, San Jose, CA, USA).

### 4.12. Measurement of IL-8/CXCL8 Secretion

Briefly, 1.5 × 10^5^ cells were infected with *S. aureus* (50 MOI) and *S. mutans* (100 MOI) for 18 h. After infection, the supernatant was collected and filtered by using 0.2 μm nylon filter. The supernatant was diluted (1:10 dilution), and IL-8/CXCL8 levels in the diluted supernatant were measured by commercially available Quantikine ELISA as per manufacturer instructions (#D8000C, R&D Systems, Toronto, ON, Canada). The detection limit of the assay is 2000–31 pg/mL. The absorbance was measured by using a Flexstation3 plate reader at the wavelength of 450 nm (Molecular Devices, San Jose, CA, USA).

## 5. Conclusions

In summary, our findings identify previously unexplored mechanisms of T2R14-mediated Gram-positive bacterial internalization and bactericidal affects in oral cells. Future studies could be focused on examining the global cellular effects of T2R14 KD and in deciphering the mechanisms involved in TLR/T2R crosstalk.

## Data Availability

The data that support the findings of this study are available on reasonable request from the corresponding author.

## Supplementary Materials

The following are available online at https://www.mdpi.com/article/10.3390/ijms22189920/s1.
