# Supplementary material for: Bitter Taste Receptor T2R14 Modulates Gram-Positive Bacterial Internalization and Survival in Gingival Epithelial Cells
Source: Int J Mol Sci. 2021 Sep 14;22(18):9920. doi: 10.3390/ijms22189920 (PMC8469602; doi:10.3390/ijms22189920)
Supplement: Supplementary file 1 [file ijms-22-09920-s001.zip › ijms-1376196-supplementary.pdf]

Supplementary Figure S1

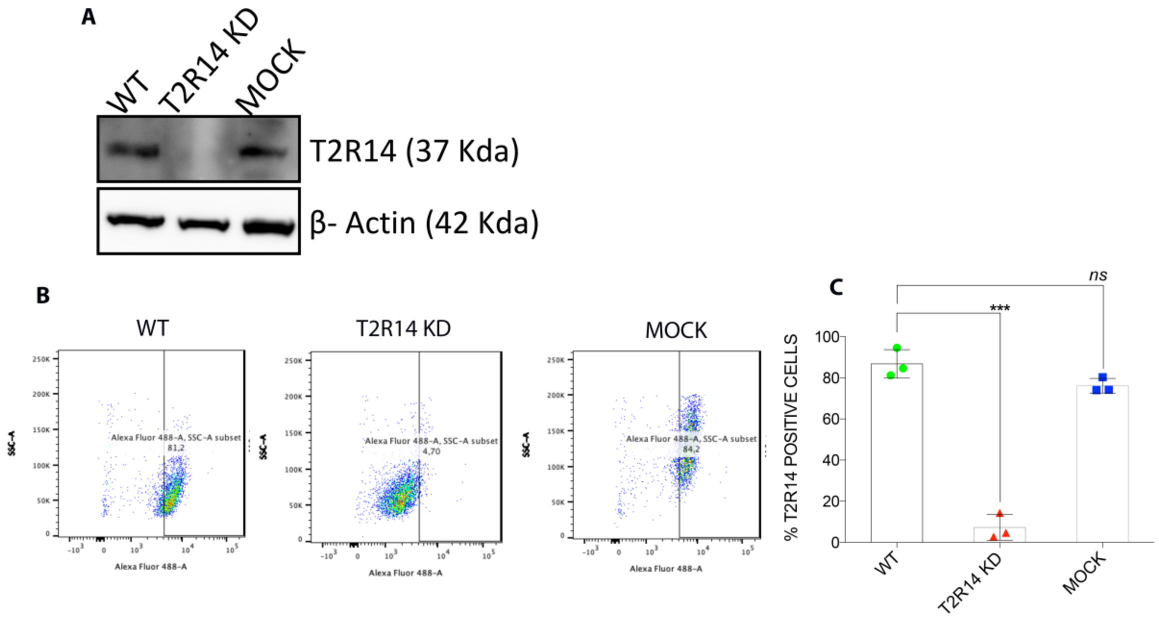

Figure S1

Figure S1. Analysis of T2R14 expression in OKF6 WT, T2R14 KD and MOCK cells.

A, Total protein extracts from the GECs were isolated using RIPA buffer and quantified using DC-protein assay kit. A total of 25 µg of protein is separated on a 10% SDS-PAGE gel and was blotted on to a PVDF membrane. The membranes were blocked with 3% skim milk and incubated with rabbit polyclonal anti-T2R14 (1:1000 dilution) and mouse monoclonal anti-β-Actin (1:25000) antibodies overnight at 4°C. The membranes were washed and incubated with secondary anti-rabbit HRP (1:3000 dilution) and anti-mouse HRP (1:20000) for 1 hour at RT. The blots were developed using chemiluminescence system and imaged using ChemiDoc MP imaging system. B, The OKF6 WT, T2R14 KD and MOCK cells were probed with rabbit polyclonal anti-T2R14 (1:300) for 2 hours and then followed with Alexa-Fluor 488 secondary anti-rabbit (1:1000) antibody. The cells were then analyzed for T2R14 cell surface expression using a BD canto analyzer. The cell populations were analyzed using flowJo software where in $T2R14^-$ population was gated on the left side of the box plot and $T2R14^+$ was gated to the right side of the box plot. C, The percentage cell populations obtained from box plots were represented in grouped bar graphs generated using graph pad prism 7.0. The data represented in the graphs are SEM of ≥ 3 independent experiments. Two-way ANOVA analysis using Tukey's multiple comparison analysis was performed and the observed *P* value is $P=0.001$.
